# Interactive Effects of Molybdenum, Zinc and Iron on the Grain Yield, Quality, and Nodulation of Cowpea (*Vigna* *unguiculata* (L.) Walp.) in North-Western India

**DOI:** 10.3390/molecules27113622

**Published:** 2022-06-05

**Authors:** Salwinder Singh Dhaliwal, Vivek Sharma, Arvind Kumar Shukla, Janpriya Kaur, Vibha Verma, Manmeet Kaur, Prabhjot Singh, Marian Brestic, Ahmed Gaber, Akbar Hossain

**Affiliations:** 1 Department of Soil Science, Punjab Agricultural University, Ferozepur Rd, Ludhiana 141027, India; ssdhaliwal@pau.edu (S.S.D.); sharmavivek@pau.edu (V.S.); janpriyakaur89@pau.edu (J.K.); vermavibha@pau.edu (V.V.); manmeetgill885@gmail.com (M.K.); prabh@pau.edu (P.S.); 2 ICAR-Indian Institute of Soil Science, Bhopal 462038, India; arvindshukla2k3@yahoo.co.in; 3 Department of Plant Physiology, Slovak University of Agriculture, Tr. A. Hlinku 2, 949 01 Nitra, Slovakia; 4 Department of Biology, College of Science, Taif University, P.O. Box 11099, Taif 21944, Saudi Arabia; a.gaber@tu.edu.sa; 5 Department of Agronomy, Bangladesh Wheat and Maize Research Institute, Dinajpur 5200, Bangladesh

**Keywords:** Cowpea, Mo soil treatment, Fe and Zn foliar application

## Abstract

Micronutrient deficiency is a major constraint for the growth, yield and nutritional quality of cowpea which results in nutritional disorders in humans. Micronutrients including molybdenum (Mo), iron (Fe) and zinc (Zn) play a pivotal role in crop nutrition, and their role in different metabolic processes in crops has been highlighted. In order to increase the nutritional quality of cowpea, a field experiment was conducted for two years in which the effect of Mo along with iron (Fe) and zinc (Zn) on productivity, nitrogen and micronutrient uptake, root length and the number of nodules in cowpea cultivation was investigated. It was found that the foliar application of Fe and Zn and their interaction with Mo application through seed priming as well as soil application displayed increased yield, nutrient concentration, uptake and growth parameters which helped to enhance the nutritional quality of cowpea for consumption by the human population. The results of the above experiments revealed that among all the treatments, the soil application of Mo combined with the foliar application of 0.5% each of FeSO_4_·7H_2_O and ZnSO_4_·7H_2_O (M_2_F_3_ treatment) enhanced the grain and stover yield of cowpea, exhibiting maximum values of 1402 and 6104.7 kg ha^−1^, respectively. Again, the M_2_F_3_ treatment resulted in higher Zn, Fe and Mo concentrations in the grain (17.07, 109.3 and 30.26 mg kg^−1^, respectively) and stover (17.99, 132.7 and 31.22 mg kg^−1^, respectively) of cowpea. Uptake of Zn, Fe and Mo by the grain (25.23, 153.3 and 42.46 g ha^−^^1^, respectively) as well as the stover (104.2, 809.9 and 190.6 g ha^−^^1^, respectively) was found to be maximum for the M_2_F_3_ treatment. The root length (30.5 cm), number of nodules per plant (73.0) and N uptake in grain and stover (55.39 and 46.15 kg ha^−^^1^) were also higher for this treatment. Overall, soil application of Mo along with the foliar application of FeSO_4_·7H_2_O (0.5%) and ZnSO_4_·7H_2_O (0.5%) significantly improved yield outcomes, concentration, uptake, root length, nodules plant^−^^1^ and N uptake of cowpea to alleviate the micronutrient deficiency.

## 1. Introduction

More than half of the world’s population consumes micronutrients at concentrations lower than their daily minimal requirements [1]. Recent data on human nutrient deficiency have shown that ‘hidden hunger’ affects more than two billion people globally [2]. Among different micronutrients, molybdenum (Mo) is also an essential trace nutrient due to its pivotal role in more than 60 enzymes that catalyze various redox reactions [3]. Its crucial role in nitrogen fixation through the enzyme nitrogenase and nitrate reductase is well known and thus affects nitrogen transport in plants [4]. On the other hand, the deficiency of Mo in crops leads to the reduced growth of flowers, smaller sizes and less maturity, consequently resulting in a lower grain yield.

Additionally, iron (Fe) and zinc (Zn) are essential nutrients for plants and their deficiencies in crops are the most common nutrient deficiencies around the globe [5]. Iron is an important structural component of numerous enzymes that are involved in various metabolic processes of plants [6]. Despite the higher abundance in the earth’s crust, its poor bioavailability in soil is due to the rapid binding with soil particles and the formation of insoluble complexes under aerobic conditions [7]. On the other hand, Zn is also a trace element that is considered crucial as it possesses antioxidant properties and is required for proper growth, immune system development, enzyme activation and neurobehavioral development [8]. A lack of Zn in the diet may result in serious health-related issues, such as stunted growth in children, increased illness susceptibility, poor birth outcomes and harm to the brain and immune system [9,10]. This has led to a growing interest in how the micronutrient content of crops might be modified in order to benefit human health and nutrition.

To improve the crop’s nutritional value, a variety of traditional interventions have been applied, including dietary supplementation, food fortification and dietary diversification [11]. Due to the lack of infrastructure, these strategies have been found to be unsuccessful. In this view, an alternate key to malnutrition named biofortification has been suggested. It is a method for enhancing the concentration of the desired mineral in a crop using specialized techniques such as plant breeding and agronomic procedures [12,13]. Further, agronomic biofortification through foliar sprays, seed priming and soil treatments are considered convenient ways to improve the nutrient content in the crop [14]. Foliar application has led to an improvement in the micronutrient status of crops, as nutrients are rapidly absorbed by the leaves at suitable growth stages [15]. On the other hand, seed priming, a pre-sowing treatment, regulates seed germination through the controlled hydration of seeds that enables the pre-germination activity. Additionally, soil treatment involves the addition of micronutrients directly into the soil which helps to pass on the available nutrients to plants for adequate plant growth.

Cowpea is a lucrative summer season vegetable and is valued for its proteins, minerals and energy [16]. Cowpea has been referred to as the poor man’s meat due to its high level of protein and is consumed by more than 200 million people from Africa, Asia and North and South America on a daily basis [17]. Besides being nutritious, it helps to sustain the productivity of the cropping systems through its ability to fix atmospheric nitrogen [18]. Poor management and inadequate cultivation on agriculturally marginal and sub-marginal lands are considered the root causes of the low productivity of pulse crops. To maximize the yield potential of pulses, the adoption of acceptable production technologies is crucial, and this can be accomplished through the use of fertilizers and micronutrients. Since Mo, Zn and Fe play key roles in the plant metabolism [19], the biofortification of these micronutrients in cowpea through the foliar application, seed priming or soil treatment would provide a potential increase in the micronutrient levels in crops, which might improve the nutritional level of crops required by humans.

Several researchers have probed the effect of enhancing bioavailable Mo, Zn and Fe in various crops through the process of biofortification. The iron biofortification of cowpea has been found to escalate the grain yield and bioavailable Fe content in cowpea [20]. Another study revealed that the seed treatment with Mo at 0.5 g/kg seed and biofertilizers in combination with the foliar application of boron significantly increased the yield attributes of cowpea [21]. The seed priming with Mo increased the grain yield and net return in chickpea [22]. However, many studies have reported the sole application of biofertilizers in cowpea [23,24] but a comparative analysis of the effect of foliar application, seed priming and soil treatment using Mo, Zn and Fe along with their interactive effects on cowpea has not been explored so far. This interactive effect could be better explored in terms of micronutrient accumulation in grain and stover along with the improvement in crop quality which could benefit human health by combating hidden hunger. The objective of the present study was to assess the influence of Mo, Zn and Fe molecules interactions on the yield, concentration and uptake of these micronutrients to enhance the food quality of crops to increase the nutritional security of the consumers.

## 2. Materials and Methods

### 2.1. Site Specification and Characteristics

The two-year field experiment was conducted at the Farm Research Area, Department of Soil Science, Punjab Agricultural University (PAU), Ludhiana, Punjab in the Indo-Gangetic plains of north-western India during the Kharif season (June–October). The experimental soil possessed a pH of 7.21, an EC of 0.34 dS m^−1^, an OC of 0.31% and had a sandy loam texture. DTPA-extractable micronutrient levels in the soil were initially 1.16 and 4.86 mg kg^−1^ for Zn and Fe, respectively. The region has a subtropical climate along with hot, rainy summers as well as dry winters. The annual rainfall ranges from 400 to 600 mm and the months of July to September receive the majority of the rainfall, which is around 70%. 

### 2.2. Treatment Details

The present experiment involved three main plot treatments of Mo application, i.e., no molybdenum (M_0_), seed priming with 500 mg kg^−1^ Mo solution (M_1_), soil application of Mo with 1.25 kg ha^−1^ (M_2_) and four subplot treatments of Zn and Fe as foliar application, i.e., no foliar spray (F_0_), 0.5% FeSO_4_·7H_2_O (F_1_), 0.5% ZnSO_4_·7H_2_O (F_2_) and 0.5% each of FeSO_4_·7H_2_O + ZnSO_4_·7H_2_O (F_3_), respectively. The details of the treatments are given in Table 1. The experiment was laid out in a split-plot design with three replications.

The recommended doses of N (19.0 kg ha^−^^1^) and P (55 kg ha^−^^1^) were applied as basal through urea and di-ammonium phosphate at the time of sowing. The sowing of the cowpea variety ‘CL 857′ was performed at 30 cm plant to plant spacing and 45 cm row to row spacing. The foliar application of FeSO_4_·7H_2_O and ZnSO_4_·7H_2_O was applied twice (at 40 days and 50 days of sowing) as per the experimental details. 

### 2.3. Harvesting and Analysis

The plants were manually harvested at the physiological maturity stage and grain as well as stover samples were collected for further analysis. Grain and stover yields were measured from the net plot area leaving the border rows and were later converted to kg ha^−1^. In order to measure the growth parameters, five plant samples were selected randomly from the central rows to measure the root length and the number of nodules. Root samples were washed with distilled water and the residual water was removed with absorbent paper. The nodules were removed quickly from the roots and counted.

To measure the dry weight, the samples were air-dried before drying in an oven at 65 °C for 48 h. A mechanical grinder was used to grind oven-dried plant samples to a fine powder. On an electric hot plate, the grounded samples of grain and stover weighing 1.0 g each were subjected to the digestion using a mixture of di-acid, i.e., HNO_3_ and HClO_4_ acid in a 3:1 ratio [25]. The micronutrient contents of Zn and Fe in digested extracts of the plant were measured using an atomic absorption spectrophotometer (Model AAS 240 FS, Company Varian, Labexchange - Die Laborgerätebörse GmbH, Bruckstr. 58, D-72393 Burladingen Germany) [26]. The samples were analyzed for N using Kjeldahl’s method [27]. Additionally, the Mo content in samples was measured by the method given by Purushottam et al. [28]. A sample (5 g) was weighed into a dry 25 mL graduated cylinder and digested with 5 mL of aqua regia for about an hour on a hot plate. After complete decomposition of the sample, 0.5 mL of phosphoric acid was added and the solution was made up to a suitable volume with demineralized water in order to determine the Mo content using AAS. The micronutrient uptake by the grain and stover of cowpea (g ha^−1^) was calculated using the following equation:

(1) $$\mathrm{Uptake}\;\left({g\;\mathrm{ha}}^{-1}\right)=\frac{\mathrm{concentration}\;\left({\mathrm{mg}\;\mathrm{kg}}^{-1}\right)\times \mathrm{Yield}\left({q\;\mathrm{ha}}^{-1}\right)}{10}$$

### 2.4. Micronutrient Use Efficiency Indices

The mobilization efficiency index (MEI) determines the translocation of nutrients towards the grain as well as the stover of crops. In the present study, the MEI calculation was performed using the following equation:

(2) $$\mathrm{MEI}=\frac{\mathrm{Nutrient}\;\mathrm{cocnentartion}\;\mathrm{in}\;\mathrm{grain}\;\left({\mathrm{mg}\;\mathrm{kg}}^{-1}\right)}{\mathrm{Nutrient}\;\mathrm{concentration}\;\mathrm{in}\;\mathrm{stover}\;\left({\mathrm{mg}\;\mathrm{kg}}^{-1}\right)}$$

The physiological efficiency (PE) indicates the increase in yield per unit of absorbed nutrient by plants and identifies the role of nutrients in increasing the crop yield. In the present study, the determination of PE of Zn, Fe and Mo viz. (PE_Zn_), (PE_Fe_), (PE_Mo_) was completed through the equations given below [29]:

(3) $$\mathrm{PE}=\frac{Y_{t}-Y_{c}}{{\mathrm{NU}}_{t}-{\mathrm{NU}}_{c}}$$

where, Y_t_ and Y_c_ denote the grain yield (kg ha^−1^) of cowpea in Mo, Zn and Fe-treated plots as well as in the control, respectively; NU_t_ and NU_c_ denote the total nutrient (Zn, Fe and Mo) uptake (kg ha^−1^) of cowpea in Mo, Zn and Fe-treated plots as well as in the control, respectively. 

### 2.5. Statistical Analysis

Data were analyzed statistically using SPSS version 16.0 (SPSS Inc., Chicago, IL, USA) packages. A two-way analysis of variance (ANOVA) and the Duncan Multiple Range test were performed to assess the significant difference between the treatment results on the crop. 

## 3. Results

The results of the study suggest that the Mo seed, as well as soil application along with a combined foliar spray of Fe and Zn, improved the yield, N and micronutrient uptake, root length and the number of nodules. The various parameters analyzed in the present study are described in the following sections.

### 3.1. Grain and Stover Yield

**Figure 1:**
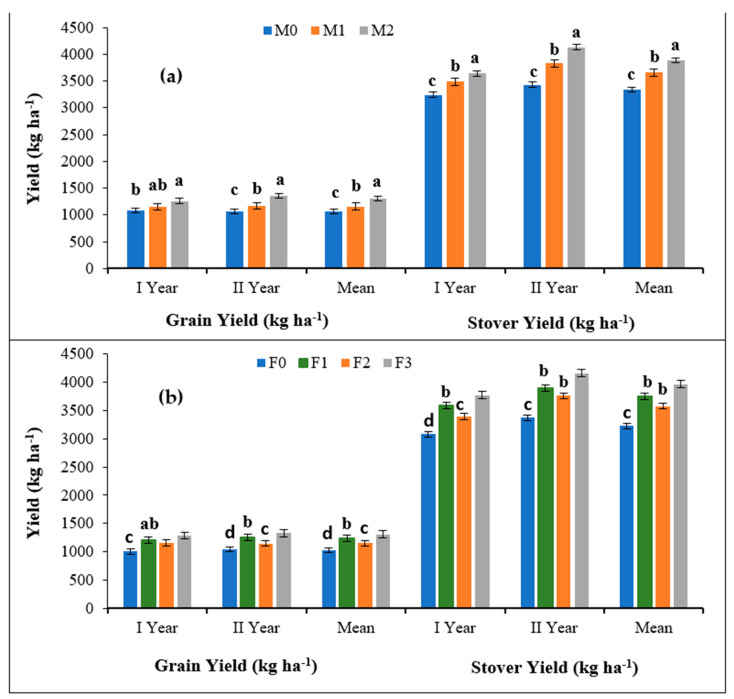
(**a**) Different methods of molybdenum and (**b**) Zn application on the grain and stover yield of cowpea over two years. The column representing the mean with a similar or dissimilar letter(s) was evaluated with the least significant difference (LSD) multiple range tests using a probability level of *p* ≤ 0.05 along with standard deviation.

The two-year mean data for Kharif 2020 and 2021 seasons demonstrated that the application of Mo, Fe and Zn had a significant effect on the grain and stover yield of cowpea (Table 2). In the main plot treatments, the average of the two-year data revealed that Mo application irrespective of the application method (M_1_ and M_2_) had a significant positive impact on the grain and stover yield (Figure 1a). Moreover, Mo application through soil (M_2_) resulted in a significantly higher grain and stover yield (1307.4 and 3886.8 kg ha^−1^) as compared to the Mo seed priming (M_1_). In sub-plot treatments, F_2_ showed a significantly higher grain and stover yield (1306.6 and 3966.5 kg ha^−1^) over the other treatments followed by F_1_, F_2_ and F_0_ (Figure 1b).

The effect of the interaction between Mo application and foliar spray further suggests that there was a significant improvement in the grain yield with the maximum value of 1402.9 kg ha^−1^ observed for the M_2_F_3_ treatment (Mo soil application + Fe + Zn foliar spray) which was statistically on par with the M_2_F_1_ treatment (Mo soil application + Fe foliar spray) and M_1_F_3_ (Mo seed application + Fe + Zn foliar spray) with grain yields of 1322.3 and 1311.6kg ha^−1^, respectively. However, the grain yield was minimum with the M_0_F_0_ treatment, i.e., the control with the mean value of 866.5 kg ha^−^^1^ which was statistically on par with M_1_F_0_ treatment, i.e., Mo seed application with no foliar spray (947.7 kg ha^−1^).

Likewise, the interactive effect of Mo as well as Fe and Zn foliar application showed that the M_2_F_3_ treatment resulted in a maximum stover yield of 6104.7 kg ha^−1^ which was statistically on par with the M_2_F_1_ treatment (3947.9 kg ha^-1^). However, the minimum value of stover yield was observed with the M_0_F_0_ treatment with a mean value of 2818.24 kg ha^−^^1^. Therefore, the results conclude that Mo soil treatment along with the combined spray of Fe and Zn significantly improved the grain as well as the stover yield of cowpea.

The effect of the interaction between Mo application and foliar spray further showed a significant improvement in grain yield with the maximum value of 1402.9 kg ha^−1^ observed for the M_2_F_3_ treatment (Mo soil application + Fe + Zn foliar spray) which was not statistically different from the M_2_F_1_ treatment (Mo soil application + Fe foliar spray) and M_1_F_3_ (Mo seed application + Fe + Zn foliar spray) with grain yields of 1322.3 and 1311.6k ha^−1^, respectively. However, the grain yield was minimum in the M_0_F_0_ treatment, i.e., the control with the mean value of 866.5 kg ha^−^^1^ which was statistically on par with the M_1_F_0_ treatment, i.e., Mo seed application with no foliar spray (947.7 kg ha^−1^).

Likewise, the interactive effect of Mo as well as Fe and Zn foliar application showed that the M_2_F_3_ treatment resulted in a maximum stover yield of 6104.7 kg ha^−1^ which was statistically on par with the M_2_F_1_ treatment (3947.9 kg ha^−1^). However, the minimum value of stover yield was observed with the M_0_F_0_ treatment with a mean value of 2818.24 kg ha^−^^1^. Therefore, the results conclude that Mo soil treatment along with the combined spray of Fe and Zn significantly improved the grain as well as the stover yield of cowpea.

### 3.2. Micronutrient Concentration in Grain and Stover

**Figure 2:**
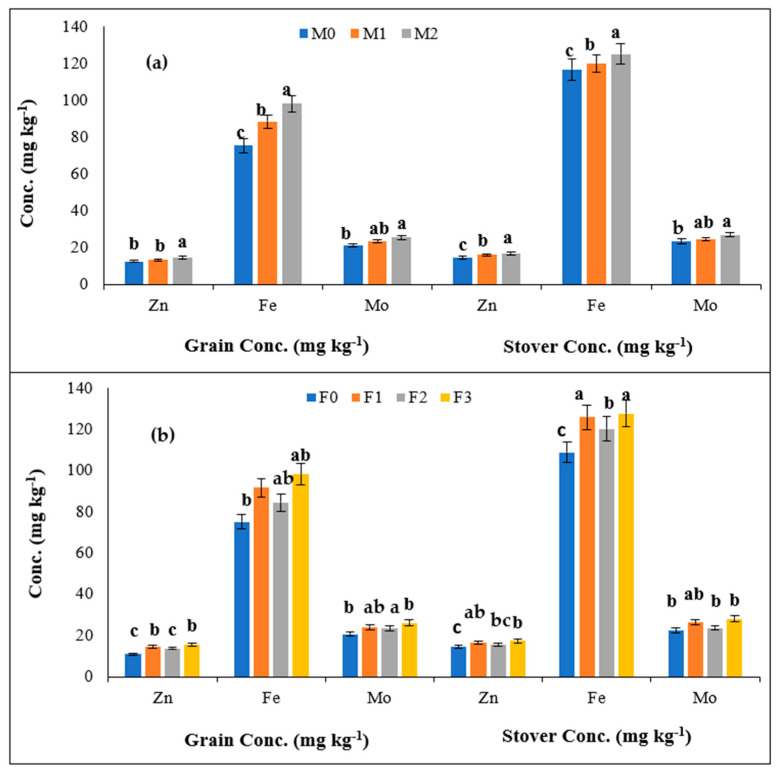
(**a**) Effect of different methods of Mo, (**b**): Fe and Zn application on Zn, Fe and Mo concentrations in the grain and stover of cowpea. Column representing the mean with a similar or dissimilar letter(s) was evaluated with the least significant difference (LSD) multiple range tests using a probability level of *p* ≤ 0.05 along with standard deviation.

The mean of two-year data pertaining to the concentrations of micronutrients in the grain and stover of cowpea is presented in Table 3. The concentration of micronutrients (Zn, Fe and Mo) in grain, as well as the stover of cowpea, increased significantly with the application of Mo either alone or in combination with the foliar application of Zn and Fe over the treatments in which no Mo, Fe and Zn were added. In the main plot treatments, the Mo application (M_1_ and M_2_) had a significant positive impact on the Zn and Fe concentrations in grain and stover (Figure 2a). Further, Mo application through the soil (M_2_) resulted in significantly higher Zn and Fe concentrations in the grain (14.72 and 98.14 mg kg^−1^) and stover (17.01 and 125.2 mg kg^−1^) as compared to the Mo seed priming (M_1_). For the Mo concentration in grain and stover, the results of M_2_ (25.66 and 27.09 mg kg^−1^) were significantly higher than M_0_, whereas the results of M_1_ (23.36 and 24.74 mg kg^−1^) were statistically on par with M_0_ (21.34 and 23.54 mg kg^−1^). In the sub-plot treatments, the F_3_ treatment resulted in the maximum Zn, Fe and Mo concentrations in grain (15.49, 98.22 and 26.05 mg kg^−1^) and stover (17.25, 127.7 and 28.09 mg kg^−1^) followed by F_1_ and F_2_, whereas minimum values were recorded in F_0_ (Figure 2b). The concentration of micronutrients (Zn, Fe and Mo) in the grain as well as the stover of cowpea, increased significantly with the application of Mo either alone or in combination with the foliar application of Zn and Fe over the treatments in which no Mo, Fe and Zn were added. 

In the main plot treatments, the Mo application (M_1_ and M_2_) had a significant positive impact on Zn and Fe concentrations in grain and stover (Figure 2a). Further, Mo application through soil (M_2_) resulted in significantly higher Zn and Fe concentrations in the grain (14.72 and 98.14 mg kg^−1^) and stover (17.01 and 125.2 mg kg^−1^) as compared to the Mo seed priming (M_1_). For the Mo concentration in grain and stover, the results of M_2_ (25.66 and 27.09 mg kg^−1^) were significantly higher than M_0_, whereas the results of M_1_ (23.36 and 24.74 mg kg^−1^) were statistically on par with M_0_ (21.34 and 23.54 mg kg^−1^). In the sub-plot treatments, the F_3_ treatment resulted in the maximum Zn, Fe and Mo concentrations in the grain (15.49, 98.22 and 26.05 mg kg^−1^) and stover (17.25, 127.7 and 28.09 mg kg^−1^) followed by F_1_ and F_2_, whereas minimum values were recorded in F_0_ (Figure 2b). Except for the Zn concentration in the grain, in all other cases, the results of F_3_ treatment were statistically on par with F_1_. The results of the interaction between Mo, Zn and Fe showed that the concentrations of Zn, Fe and Mo ranged from 9.94 to 17.07, 62.55 to 109.3 and 18.44 to 30.26 mg kg^−^^1^, respectively, in the grain of cowpea under different treatments. The interactive effects show that the M_2_F_3_ treatment involving Mo soil treatment along with combined foliar Fe and Zn application showed maximum concentrations of Zn, Fe and Mo (17.07, 109.3 and 30.26 mg kg^−^^1^) as compared to the M_0_F_0_ treatment with concentration values of 9.94, 62.55 and 18.44 mg kg^−^^1^, respectively. Moreover, in the case of Fe, the results of the M_2_F_3_ treatment were found to be statistically on par with the M_2_F_1_ and M_2_F_2_ treatments (105.6 and 93.86 mg kg^−^^1^, respectively). Additionally, for Mo, its concentration with the M_2_F_3_ treatment (30.26 mg kg^−^^1^) was not statistically different from the M_2_F_1_ treatment (26.06 mg kg^−1^). In the case of stover, the interactive studies between Mo, Fe and Zn applications show that the maximum increase in micronutrient level was observed in the M_2_F_3_ treatment with concentrations of 17.99, 132.7 and 31.22 mg kg^−^^1^ for Zn, Fe and Mo, respectively. However, the M_0_F_0_ treatment revealed the minimum micronutrient concentrations of 12.72, 103.9, and 21.50 mg kg^−^^1^, respectively. In case of Zn, the M_2_F_3_ treatment was statistically on par with the M_2_F_1_, M_2_F_2_, M_1_F_3_ and M_0_F_3_ treatments (17.19, 16.81, 17.20 and 16.57 mg kg^−^^1^, respectively). For Fe, the results of the M_2_F_3_ treatment were not statistically different from the M_2_F_1_ treatment (130.5 mg kg^−^^1^). Similarly, for Mo, its concentration in the M_2_F_3_ treatment (31.22 mg kg^−^^1^) was not statistically different from the M_2_F_1_, M_1_F_3_, M_1_F_1_ and M_0_F_3_ treatments (28.40, 27.09, 26.20 and 25.90 mg kg^−^^1^).

### 3.3. Micronutrient Uptake by Grain and Stover 

**Figure 3:**
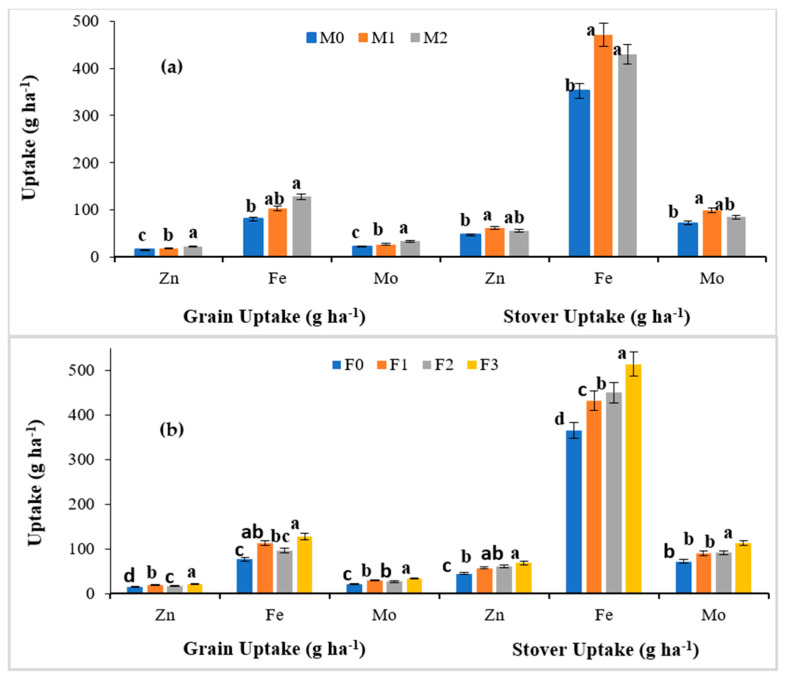
(**a**) Effect of different methods of Mo and (**b**) Fe and Zn application on Zn, Fe and Mo uptake in the grain and stover of cowpea. The column representing the mean with a similar or dissimilar letter(s) was evaluated with the least significant difference (LSD) multiple range tests using a probability level of *p* ≤ 0.05 along with standard deviation.

The uptake of micronutrients increased significantly in both grain as well as the stover of cowpea with the combined application of Mo, Fe and Zn over the treatments in which no or sole application of Mo, Fe and Zn was carried out (Table 4). In the main plot treatments, the highest increase in micronutrient uptake was observed in the M_2_ treatment among the different Mo applications with uptake values of 22.24, 128.3 and 33.55 g ha^−1^ for Zn, Fe and Mo, respectively, in the grain of cowpea (Figure 3a). The results of Fe uptake in the M_2_ treatment were statistically on par with the M_1_ treatment (102.6 g ha^−1^). 

In the case of stover uptake in cowpea, M_2_ treatment, i.e., soil Mo application led to the highest micronutrient uptakes of 55.52, 430.6 and 84.76 g ha^−1^ for Zn, Fe and Mo, respectively. Moreover, the results of the M_2_ treatment were statistically on par with the M_1_ treatment with uptake values of 61.76, 471.4 and 99.10 g ha^−1^ for Zn, Fe and Mo, respectively. In the subplot treatments, the F_3_ treatment showed a higher improvement in micronutrient uptakes with values of 22.55, 128.3 and 34.04 g ha^−^^1^ for Zn, Fe and Mo, respectively (Figure 3b). Moreover, the Zn, Fe and Mo uptake in the stover of cowpea were recorded as the highest in F_3_ as compared to no or the sole application of Fe and Zn (68.94, 513.6 and 113.29 g ha^−1^, respectively). The effect of the interaction between Mo, Fe and Zn suggests that the M_2_F_3_ treatment showed the maximum micronutrient uptake in the grain of cowpea with values of 25.23, 153.3 and 42.46 g ha^−^^1^ for Zn, Fe and Mo, respectively. In the case of Fe, the results of the M_2_F_3_ treatment were not statistically different from the M_2_F_1_ (139.7 g ha^−1^) and M_1_F_3_ (126.2 g ha^−1^) treatments. However, the minimum uptake of 11.02, 54.20 and 15.98 g ha^−1^ for the Zn, Fe and Mo micronutrients was observed in the M_0_F_0_ treatment, i.e., the control. 

In the case of Zn, Fe and Mo uptake in stover, the combined use of Mo soil treatment along with Fe and Zn foliar application, i.e., M_2_F_3_ treatment, further enhanced the Zn, Fe and Mo uptake to 104.2, 809.9 and 190.6 g ha^−1^, respectively. The results of this treatment were statistically on par with the M_2_F_1_ (88.29 g ha^−1^) and M_1_F_3_ (88.66 g ha^−1^) treatments in the case of Zn. Similarly for Fe, the M_2_F_3_ treatment was not statistically different from the M_2_F_1_ treatment with values of 757.4 g ha^−1^. However, the minimum uptake of 41.24, 431.5 and 89.21 g ha^−1^ for Zn, Fe and Mo micronutrients was observed in the M_0_F_0_ treatment, i.e., the control. 

### 3.4. Root Length, Nodules and N Uptake

The results of two years’ mean data concerning the root length, the number of nodules plant^−1^ and N concentration in grain as well as the stover of cowpea are given in Table 5. 

**Figure 4:**
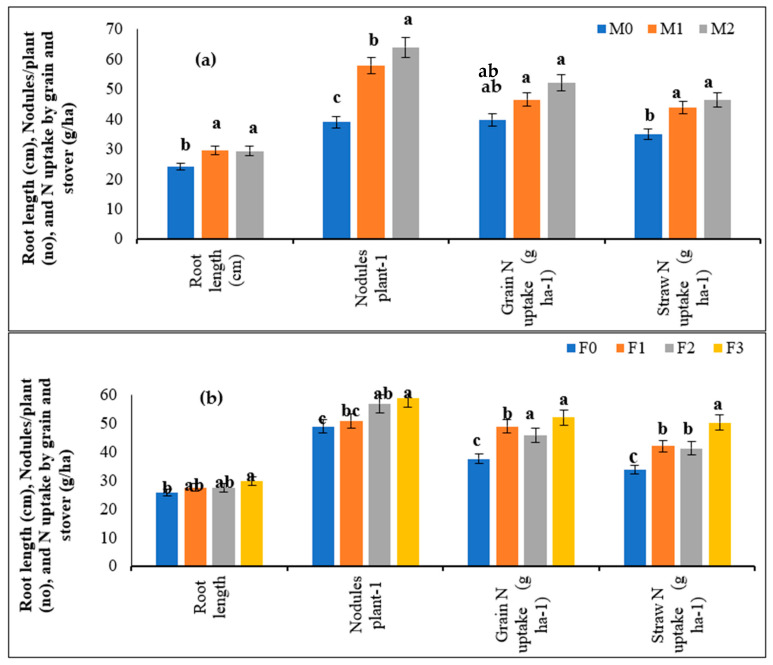
(**a**) Effect of different methods of Mo and (**b**) Fe and Zn application on root length, nodules plant^−1^, N uptake in grain and stover yield of cowpea. The column representing the mean with a similar or dissimilar letter(s) was evaluated with the least significant difference (LSD) multiple range tests using a probability level of *p* ≤ 0.05 along with standard deviation.

In the main plot treatments (Figure 4a), the root length of cowpea increased significantly with Mo application over the control (24.1 cm). The results of seed priming of Mo (29.5 cm) were statistically on par with the soil application of Mo (29.5 cm). Moreover, the number of plant^-1^ nodules increased significantly with Mo application over the control (39.0). Moreover, the results under soil application (64.0) were significantly higher over the seed priming of Mo (58.0). The N uptake increased significantly with the Mo application over the control in the grain and stover of cowpea (39.75 and 35.07 kg ha^−1^, respectively). 

The results of the seed-primed Mo treatment (M_1_) were statistically on par with the soil application of Mo treatment (M_2_). In the sub-plot treatment (Figure 4b), the root length also increased significantly with the foliar application of Fe+Zn (29.8 cm) over the control (25.9 cm). The number of plant^−1^ nodules was significantly higher with the combined application of Fe+Zn (59.0), i.e., F_3_ over the F_0_ (49.0), F_1_ (51.0) and F2 (57.0). For grain N uptake, the highest values were obtained with the combined application of Fe+Zn (52.20 kg ha^−1^) under the F_3_ treatment, which was significantly higher over the F_0_ (37.56 kg ha^−1^). A similar trend was observed for stover N uptake with the foliar application of Fe and Zn. Under the interactive effects, the root length showed a non-significant variation. The highest number of plant^−1^ nodules in cowpea were recorded in M_2_F_3_ (73.0), whereas the lowest value was recorded in M_0_F_0_ (31.0). The results of M_2_F_3_ were statistically on par with the M_2_F_2_ treatment (69.0). The interactive effects of Mo, Fe and Zn also showed significant effects on N uptake in the grain and stover. The maximum N uptake in grain was recorded under the M_2_F_1_ treatment (55.91 kg ha^−1^), whereas the minimum value was observed under the M_0_F_0_ treatment (29.36 kg ha^−1^). The results of M_2_F_1_ were statistically at par with M_1_F_3_ and M_2_F_3_. The data for stover N uptake showed that the highest results were obtained under the M_1_F_1_ treatment (56.20 kg ha^−1^) and the lowest value was recorded under the M_0_F_0_ treatment (22.07 kg ha^−1^). 

### 3.5. Efficiency Indices

The results of Table 6 demonstrate that MEI-Zn was at a maximum with the M_2_F_3_ treatment (0.949) showing the soil-applied Mo along with Fe and Zn foliar application and was lowest in the M_1_F_0_ treatment (0.726). Similarly, for Fe and Mo, MEI was highest in the M_2_F_3_ treatment with values of 0.824 and 0.969, respectively and was lowest in the M_0_F_0_ treatment for Fe (0.602), and M_1_F_2_ for Mo (0.708). Additionally, the results of PE-Zn, PE-Fe and PE-Mo were highest in the M_2_F_0_ treatment with values of 0.572 q/g, 0.069 q/g and 0.350 q/g, respectively. However, the lowest PE values were observed in the M_2_F_3_ treatment (0.323 q/g) for Zn, M_2_F_1_ (0.051 q/g) for Fe and M_2_F_3_ (0.195 q/g) for Mo, respectively. 

## 4. Discussion

### 4.1. Grain and Stover Yield

The results displayed in Table 2 and Figure 1 reveal that the grain as well as the stover yield of cowpea significantly increased for two years and was the maximum for M_2_ treatment among the Mo treatments. Thus, the presence of Mo in the M_1_ and M_2_ treatments improved the grain as well as the stover yield as compared to the control. The trend might be attributed to the vital role of Mo in the synthesis and activity of molybdoenzymes which regulates the N fixation, thus increasing the N content and crop yield [30]. The improved yield with Mo application could also be ascribed to its outstanding role in photosynthesis and respiration processes. In the absence of molybdenum in soil, the plant molybdoenzymes could break and adversely affect the nitrogen fixation by soil bacteria which results in a reduced yield [31]. Additionally, the higher yield observed in the foliar Fe application as compared to Zn was largely related to the crucial role of Fe in the synthesis of growth promoters such as auxins, photosynthesis, seed maturation and nucleic acid metabolism which results in a significantly higher grain and stover yield [32,33]. However, Zn foliar application reduced the yield attributes which may have been due to the lower macronutrient concentrations in the grain.

Additionally, the double and triple micronutrient application exhibited superior grain and stover yields over single micronutrients which might have been due to the synergistic interactions involved among Mo, Fe and Zn. Combined soil treatment with Mo along with the foliar application of Fe had a favorable effect on nitrogenase activity in nodules and nitrate reductase activity in the plant system. Studies in the literature have reported that the application of essential nutrients (N, Fe and Mo) at the optimum level positively influences the metabolic processes and thus leads to a higher yield of cowpea. The application of Mo along with micronutrients and Rhizobium inoculation has also recorded enhanced cowpea growth and nodulation [34]. Another study reported that the seed treatment with Mo solution can overcome the internal Mo deficiencies and thus maintain the activity of molybdoenzymes [35]. Similarly, a significant effect on the root growth and yield of soybean has been observed under the seed inoculation with Rhizobium and Mo [36]. 

### 4.2. Micronutrient Concentrations in the Grain and Stover 

The data (Table 3 and Figure 2) revealed that the sole and combined application of Mo, Fe and Zn led to a significant improvement in the micronutrient concentrations in cowpea grain and stover as compared to control which might have been due to the immediate absorption of available micronutrients by plant leaves. Among the Mo treatments, the M_2_ treatment proved beneficial for enhancing the micronutrient concentration, where Mo played a major role in the functioning of nitrate and nitrite reductase [31]. The present findings are concordant with previous studies, where Mo supplementation increased the Mo concentration in ‘*Le-Conte*’ pear [37], grapes (cv. Merlot) [38], peanut [39] and lettuce [40]. Togay et al. [41] also reported the enhanced concentration of Fe, P, Mn, Cu and Mo in lentil (*Lens culinaris* Medic.) through the combined Fe (20 kg ha^−1^) and Mo (6 g kg^−1^ seed) application. Our findings are in line with the aforementioned studies, suggesting that the application of Mo increased the concentration of Zn, Fe and Mo in the grain and stover of cowpea.

However, the foliar application of Fe+Zn in the F_3_ treatment also resulted in an increased micronutrient concentration in cowpea as compared to other treatments including the control, i.e., F_0_. This might have been due to the higher availability of these micronutrients to the crop at the optimum level of application [13]. Another important point is the interaction between micronutrients which affects their uptake, distribution and utilization in plants [42]. Additionally, the combined application of Mo through soil treatment along with the Fe and Zn foliar spray further increased the micronutrient levels in both the grain and stover of cowpea; thus, it could be inferred that Mo, Fe and Zn possessed the appropriate mechanisms for the translocation of micronutrients to the grain and stover in cowpea. The enhancement in the nutrient content might have been due to an increased absorption as well as the assimilation of the micronutrients that resulted in balanced nutritional value in the crop for higher growth and thereby a higher nutrient content. Similar results were observed by Hristozkova et al. [43] where Mo enhanced the accumulation of nutrients in cowpea plant tissues. Gad and Kandil [44] added that the presence of Mo and N significantly increased the composition of minerals such as N, P, K, Fe, Mn, Zn, Cu and Mo in cowpea with all nitrogen levels as compared to the untreated plants.

### 4.3. Micronutrient Uptake by the Grain and Stover 

The results of the present study (Table 4 and Figure 3) demonstrated that micronutrient uptake was found to increase significantly with external supplementation. The trend could be coupled with the joint impact of yield as well as concentration. Moreover, the exogenous supply of nutrients through the treatment with Mo, Fe and Zn molecules increased the availability of these nutrients in the soil to a remarkable extent. The results in the present study are in agreement with previous studies in which the application of Mo resulted in an improved Fe, P, Mn and Mo uptake in rice [45]. Similarly, Ndakidemi et al. [46] suggested a significant increase in Mo uptake (0.644 mg plant^−1^) in the roots of the common bean (*Phaseolus vulgaris* L.) with the Mo application at 12 g kg^−1^ as compared to the control. This could be attributed to the absorption of an increased quantity of Mo from the soil which led to its improved concentration and uptake in the common bean. Overall, the combined application of Mo, Fe and Zn molecules was most effective in increasing the micronutrient uptake in the grain and stover of cowpea.

### 4.4. Root Length, Nodules and N Concentration of Cowpea

The present findings indicate the beneficial effects of Mo application on root length as with Mo application there was a significant increase in root length of cowpea (Table 5 and Figure 4). The direct effect of Mo on root growth has not been reported yet; however, the results might be attributed to the enhanced activity of various enzymes, such as nitrogenase and nitrate reductase, the growth of root nodules and the promotion of the hormone synthesis to be transported in roots [47,48]. Similar results have been reported by Liu et al. [49] in which Mo application enhanced the root length of soybean. Additionally, Mo also plays a crucial role in N metabolism through Mo enzymes and plays a key role in carrying out redox reactions [8,31]. The trend of the number of nodules and nitrogen uptake can also be explained based on the above reasons. The Mo cofactor ‘FeMoCo’ increases the activity of enzymes involved in nitrogen fixation that catalyzes the inorganic nitrogen assimilation. After the uptake by roots, the nitrates are directed towards the plant vacuoles. In plants, nitrate is reduced to ammonium (NH_4_^+^) through an enzymatic reaction. Initially, the transformation of NO_3_^−^ to NO_2_^−^ occurs in the cytoplasm in the presence of nitrate reductase followed by its conversion in NO_4_^+^ in proplastids or chloroplasts catalyzed by nitrite reductase [50]. The results of the present study have demonstrated the significant association of N accumulation with soil Mo application. The absence of Mo promotes nitrate accumulation and indicates less N assimilation by the plants [40]. Similar results have been reported in alfalfa nodulation with Mo supplementation [51]. 

### 4.5. Efficiency Indices of Cowpea

The results of MEI indicated that the MEI of Mo was higher in the presence of the Mo soil treatment along with Fe and Zn foliar application (Table 6). On the other hand, the results of PE indicated an increase in grain production with the absorbed nutrient. The higher values for PE-Zn, PE-Fe and PE-Mo were found in the M_2_F_3_ treatment involving Mo soil application along with Fe and Zn foliar application as compared to no or sole applications of Mo, Fe and Zn.

## 5. Conclusions

The findings of a two-year study clarified that the supplementation of Mo, Fe and Zn molecules through ammonium molybdate, FeSO_4_·7H_2_O and ZnSO_4_·7H_2_O influenced the yield, quality and root system of cowpea. The treatment involving Mo soil application along with a foliar spray of FeSO_4_·7H_2_O (0.5%) + ZnSO_4_·7H_2_O (0.5%) resulted in the highest increased yield, micronutrient concentration and uptake in cowpea. The root length and the number of nodules were also enhanced with the Mo application. The presence of Mo and Fe molecules enhanced the N content and helped in nitrogen fixation which in turn improved the nutritional quality of the produce. Additionally, the efficiency indices, i.e., MEI and PE were maximum in the treatment involving Mo application along with the foliar spray of Fe and Zn. Thus, Mo soil treatment along with Fe and Zn application could be considered the most efficient strategy for enhancing the grain and stover yield along with the availability of micronutrients for improved cultivation of cowpea.

## Figures and Tables

**Table 1:** Treatment details of the field experiment.

Treatments	Details
M_0_F_0_	Control
M_0_F_1_	0.5% FeSO_4_·7H_2_O
M_0_F_2_	0.5% ZnSO_4_·7H_2_O
M_0_F_3_	0.5% FeSO_4_·7H_2_O + 0.5% ZnSO_4_·7H_2_O
M_1_F_0_	Mo seed priming
M_1_F_1_	Mo seed priming + 0.5% FeSO_4_·7H_2_O
M_1_F_2_	Mo seed priming + 0.5% ZnSO_4_·7H_2_O
M_1_F_3_	Mo seed priming + 0.5% FeSO_4_·7H_2_O + 0.5% ZnSO_4_·7H_2_O
M_2_F_0_	Mo soil application
M_2_F_1_	Mo soil application + 0.5% FeSO_4_·7H_2_O
M_2_F_2_	Mo soil application + 0.5% ZnSO_4_·7H_2_O
M_2_F_3_	Mo soil application + 0.5% FeSO_4_·7H_2_O + 0.5% ZnSO_4_·7H_2_O

**Table 2:** Effect of different levels of Zn, Fe and Mo molecules on the grain and stover yield of cowpea.

Treatments	Grain Yield (kg ha^−1^)			Stover Yield (kg ha^−1^)		
Interaction	I Year	II Year	Mean	I Year	II Year	Mean
M_0_F_0_	884.7 ^f^ ± 153.3	848.2 ^f^ ± 49.9	866.5 ^f^ ± 25.8	2662.4 ^h^ ± 112.2	2973.9 ^e^ ± 20.0	2818.2 ^g^ ± 65.2
M_0_F_1_	1138.8 ^de^ ± 84.1	1175.9 ^cd^ ± 23.6	1157.4 ^d^ ± 26.3	3490.0 ^cde^ ± 123.4	3502.8 ^d^ ± 50.6	3496.4 ^e^ ± 51.5
M_0_F_2_	1089.9 ^e^ ± 46.5	1022.2 ^e^ ± 90.8	1056.1 ^e^ ± 47.8	3150.25 ^g^ ± 168.6	3280.8 ^d^ ± 154.2	3215.5 ^f^ ± 10.2
M_0_F_3_	1206.7 ^bcd^ ± 31.6	1204.2 ^cd^ ± 76.5	1205.4 ^cd^ ± 53.3	3676.5 ^bc^ ± 89.8	3969.6 ^bc^ ± 186.7	3823.0 ^bc^ ± 68.5
M_1_F_0_	943.8 ^f^ ± 40.3	951.7 ^ef^ ± 72.3	947.7 ^f^ ± 55.8	3231.2 ^fg^ ± 258.9	3279.1 ^d^ ± 169.1	3255.1 ^f^ ± 63.5
M_1_F_1_	1201.9 ^bcd^ ± 49.4	1251.3 ^bc^ ± 120.8	1226.6 ^bcd^ ± 34.8	3574.5 ^bcde^ ± 244.3	4001.9 ^c^ ± 114.9	3788.2 ^bcd^ ± 91.4
M_1_F_2_	1160.1 ^de^ ± 40.1	1138.7 ^d^ ± 137.7	1149.4 ^e^ ± 15.1	3393.8 ^ef^ ± 506.6	3969.4 ^bc^ ± 94.1	3681.6 ^cde^ ± 291.7
M_1_F_3_	1299.9 ^ab^ ± 181.9	1323.1 ^b^ ± 50.4	1311.6 ^ab^ ± 16.4	3756.9 ^ab^ ± 203.8	4067.7 ^bc^ ± 198.8	3912.3 ^b^ ± 30.6
M_2_F_0_	1180.2 ^cde^ ± 70.6	1334.7 ^b^ ± 90.7	1257.5 ^bc^ ± 109.2	3346.2 ^cf^ ± 115.8	3856.4 ^c^ ± 114.2	3601.3 ^de^ ± 42.3
M_2_F_1_	1286.4 ^abc^ ± 97.5	1358.2 ^ab^ ± 87.3	1322.3 ^ab^ ± 50.8	3709.3 ^ab^ ± 86.1	4186.6 ^b^ ± 339.5	3947.9 ^ab^ ± 179.2
M_2_F_2_	1222.4 ^bcd^ ± 125.8	1271.5 ^bc^ ± 70.8	1246.9 ^bcd^ ± 34.7	3634.8 ^bcd^ ± 186.7	4032.7 ^bc^ ± 129.9	3833.7 ^bc^ ± 40.2
M_2_F_3_	1350.2 ^a^ ± 102.2	1455.6 ^a^ ± 93.8	1402.9 ^a^ ± 74.4	3880.7 ^a^ ± 188.4	4447.9 ^a^ ± 411.4	4164.3 ^a^ ± 157.7
LSD (_0.05_)	112.2	107.4	97.2	187.5	245.2	209.4

M_0_: No molybdenum, M_1_: Molybdenum seed priming, M_2_: Molybdenum soil treatment, F_0_: No fertilizer application, F_1_: Fe application, F_2_: Zn application, F_3_: Fe+Zn application. The mean with a similar or dissimilar letter(s) was evaluated with the least significant difference (LSD) multiple range tests using a probability level of *p* ≤ 0.05 along with standard deviation.

**Table 3:** Effect of the application of Zn, Fe and Mo molecule on the concentration of micronutrients in the grain and stover of cowpea.

Treatments	Grain Concentration (mg kg^−1^)			Stover Concentration (mg kg^−1^)		
Interaction	Zn	Fe	Mo	Zn	Fe	Mo
M_0_F_0_	9.94 ^g^ ± 6.1	62.55 ^e^ ± 21.7	18.44 ^d^ ± 2.0	12.72 ^f^ ± 0.7	103.9 ^g^ ± 5.3	21.50 ^d^ ± 3.8
M_0_F_1_	13.65 ^de^ ± 5.3	78.46 ^cde^ ± 37.6	21.98 ^bcd^ ± 6.9	15.74 ^bcd^ ± 2.4	122.8 ^cd^ ± 0.6	24.58 ^bd^ ± 0.7
M_0_F_2_	12.82 ^ef^ ± 4.7	71.56 ^de^ ± 30.8	22.31 ^bcd^ ± 8.9	13.47 ^ef^ ± 3.9	116.2 ^e^ ± 3.7	22.12 ^cd^ ± 4.7
M_0_F_3_	14.11 ^cd^ ± 5.5	89.10 ^bc^ ± 54.8	22.64 ^bcd^ ± 1.1	16.57 ^abc^ ± 2.7	124.1 ^bc^ ± 1.1	25.9 ^abcd^ ± 1.9
M_1_F_0_	10.74 ^g^ ± 7.8	78.87 ^cd^ ± 37.3	20.71 ^c^ ± 2.9	14.79 ^de^ ± 0.2	108.37 ^f^ ± 2.6	22.46 ^cd^ ± 2.3
M_1_F_1_	14.08 ^cd^ ± 7.4	90.95 ^bc^ ± 46.4	24.07 ^bc^ ± 6.2	16.47 ^bc^ ± 2.6	124.0 ^bc^ ± 8.5	26.20 ^abcd^ ± 0.4
M_1_F_2_	13.55 ^de^ ± 7.1	88.02 ^bc^ ± 44.2	23.40 ^bc^ ± 12.0	15.98 ^bcd^ ± 2.9	120.9 ^d^ ± 3.3	23.20 ^bcd^ ± 2.1
M_1_F_3_	15.31 ^b^ ± 6.6	96.22 ^ab^ ± 54.1	25.24 ^b^ ± 5.5	17.20 ^ab^ ± 1.9	126.4 ^b^ ± 3.7	27.09 ^abc^ ± 5.2
M_2_F_0_	11.84 ^f^ ± 7.2	83.66 ^bcd^ ± 43.6	22.23 ^bcd^ ± 6.8	16.05 ^cd^ ± 0.8	114.2 ^e^ ± 6.8	23.24 ^bcd^ ± 0.2
M_2_F_1_	15.22 ^b^ ± 7.7	105.6 ^a^ ± 45.7	26.06 ^ab^ ± 10.7	17.19 ^ab^ ± 3.3	130.5 ^a^ ± 2.1	28.40 ^ab^ ± 8.0
M_2_F_2_	14.77 ^bc^ ± 7.4	93.86 ^abc^ ± 42.1	24.10 ^b^ ± 9.2	16.81 ^abc^ ± 3.5	123.4 ^cd^ ± 0.9	25.50 ^bcd^ ± 1.4
M_2_F_3_	17.07 ^a^ ± 6.8	109.3 ^a^ ± 64.4	30.26 ^a^ ± 3.7	17.99 ^a^ ± 3.7	132.7 ^a^ ± 1.4	31.22 ^a^ ± 3.4
LSD (0.05)	1.0	16.2	4.2	1.5	2.9	5.5

M_0_: No molybdenum, M_1_: Molybdenum seed priming, M_2_: Molybdenum soil treatment, F_0_: No fertilizer application, F_1_: Fe application, F_2_: Zn application, F_3_: Fe+Zn application. The mean with a similar or dissimilar letter(s) was evaluated with the least significant difference (LSD) multiple range tests using a probability level of *p* ≤ 0.05 along with standard deviation.

**Table 4:** Effect of the application of Zn, Fe and Mo molecules on the uptake of micronutrients in the grain and stover of cowpea.

Treatments	Uptake in Grain (g ha^−1^)			Uptake in Stover (g ha^−1^)		
	Zn	Fe	Mo	Zn	Fe	Mo
M_0_F_0_	11.02 ^f^ ± 5.6	54.20 ^g^ ± 17.2	15.98 ^g^ ± 0.4	35.85 ^g^ ± 18.4	292.81 ^g^ ± 158.7	60.59 ^d^ ± 20.1
M_0_F_1_	18.22 ^d^ ± 5.7	90.81 ^def^ ± 45.6	25.44 ^ef^ ± 4.5	55.03 ^cde^ ± 41.8	429.35 ^cd^ ± 304.1	85.94 ^bc^ ± 56.7
M_0_F_2_	14.22 ^e^ ± 5.6	75.57 ^efg^ ± 29.1	23.56 ^f^ ± 3.8	43.31 ^ef^ ± 39.5	373.64 ^ef^ ± 236.4	71.12 ^d^ ± 69.5
M_0_F_3_	19.98 ^cd^ ± 6.7	107.4 ^cd^ ± 65.9	27.29 ^def^ ± 0.7	63.34 ^bc^ ± 43.8	474.43 ^bc^ ± 296.4	99.01 ^b^ ± 73.1
M_1_F_0_	13.99 ^e^ ± 7.3	74.75 ^fg^ ± 35.8	19.63 ^g^ ± 1.5	48.14 ^fg^ ± 24.7	352.75 ^f^ ± 243.9	73.10 ^cd^ ± 61.7
M_1_F_1_	20.19 ^c^ ± 8.5	111.6 ^bc^ ± 60.1	29.53 ^ce^ ± 4.6	62.39 ^bcd^ ± 40.7	469.73 ^bc^ ± 306.4	99.25 ^b^ ± 56.0
M_1_F_2_	18.37 ^d^ ± 8.4	101.1 ^cdef^ ± 49.5	26.89 ^def^ ± 6.6	58.83 ^bcde^ ± 36.9	445.10 ^cd^ ± 229.6	85.41 ^c^ ± 35.2
M_1_F_3_	22.56 ^b^ ± 8.4	126.2 ^abc^ ± 72.4	33.09 ^bc^ ± 4.0	67.39 ^ab^ ± 44.7	494.51 ^b^ ± 321.9	105.98 ^ab^ ± 96.1
M_2_F_0_	20.19 ^c^ ± 7.2	105.2 ^cde^ ± 63.9	27.95 ^de^ ± 6.4	57.80 ^def^ ± 21.0	411.26 ^de^ ± 206.2	83.69 ^cd^ ± 47.8
M_2_F_1_	22.74 ^b^ ± 9.3	139.7 ^ab^ ± 65.9	34.46 ^b^ ± 8.2	67.88 ^ab^ ± 46.9	515.20 ^ab^ ± 298.3	112.12 ^ab^ ± 111.4
M_2_F_2_	20.97 ^bc^ ± 8.6	117.0 ^bcd^ ± 55.8	30.05 ^cd^ ± 6.5	64.44 ^bc^ ± 46.5	473.07 ^bc^ ± 282.4	97.75 ^bc^ ± 66.4
M_2_F_3_	25.23 ^a^ ± 8.1	153.3 ^a^ ± 98.6	42.46 ^a^ ± 0.3	74.54 ^a^ ± 54.6	552.60 ^a^ ± 310.8	130.00 ^a^ ± 93.7
LSD (0.05)	1.8	30.0	4.3	11.1	47.0	24.7

M_0_: No molybdenum, M_1_: Molybdenum seed priming, M_2_: Molybdenum soil treatment, F_0_: No fertilizer application, F_1_: Fe application, F_2_: Zn application, F_3_: Fe+Zn application. The mean with a similar or dissimilar letter(s) was evaluated with the least significant difference (LSD) multiple range tests using a probability level of *p* ≤ 0.05 along with standard deviation.

**Table 5:** Effect of the application of Zn, Fe and Mo molecules on root length, number of nodules and N concentration of cowpea.

Treatments	Root Length (cm)	Nodules Plant^−1^ (no)	N Uptake (kg ha^−1^)	
Molybdenum			Grain	Stover
Interaction				
M_0_F_0_	20.3 ± 2.5	31.0 ^f^ ± 1.5	29.36 ^f^ ± 2.2	22.07 ^f^ ± 5.4
M_0_F_1_	23.5 ± 3.2	34.5 ^ef^ ± 2.2	40.83 ^d^ ± 2.1	39.62 ^de^ ± 2.6
M_0_F_2_	24.9 ± 0.5	44.0 ^de^ ± 4.6	41.69 ^d^ ± 0.5	36.22 ^e^ ± 4.8
M_0_F_3_	27.7 ± 2.1	47.0 ^d^ ± 3.2	47.93 ^cd^ ± 0.8	44.38 ^bcd^ ± 2.5
M_1_F_0_	28.3 ± 2.5	62.0 ^bc^ ± 3.7	36.62 ^e^ ± 1.0	34.10 ± 1.9
M_1_F_1_	28.5 ± 4.1	56.0 ^cd^ ± 1.2	50.83 ^bc^ ± 2.1	56.20 ^a^ ± 1.0
M_1_F_2_	29.8 ± 2.0	57.0 ^cd^ ± 1.0	46.02 ^d^ ± 3.,2	43.67 ^bcd^ ± 2.0
M_1_F_3_	31.3 ± 0.4	57.0 ^cd^ ± 1.5	53.25 ^ab^ ± 2.5	42.16 ^cd^ ± 2.2
M_2_F_0_	29.3 ± 0.9	53.0 ^cde^ ± 0.8	47.19 ^cd^ ± 4.7	47.26 ^b^ ± 3.4
M_2_F_1_	30.5 ± 1.0	62.0 ^bc^ ± 3.1	55.91 ^a^ ± 3.1	47.12 ^b^ ± 3.6
M_2_F_2_	27.5 ± 2.5	69.0 ^ab^ ± 2.3	49.93 ^bc^ ± 2.0	44.30 ^bcd^ ± 0.6
M_2_F_3_	30.5 ± 2.3	73.0 ^a^ ± 1.0	55.39 ^a^ ± 1.0	46.15 ^bc^ ± 2.7
LSD (0.05)	NA	10.5	3.54	4.87

M_0_: No molybdenum, M_1_: Molybdenum seed priming, M_2_: Molybdenum soil treatment, F_0_: No fertilizer application, F_1_: Fe application, F_2_: Zn application, F_3_: Fe+Zn application. The mean with a similar or dissimilar letter(s) was evaluated with the least significant difference (LSD) multiple range tests using a probability level of *p* ≤ 0.05 along with standard deviation.

**Table 6:** Effect of the application of Zn, Fe and Mo molecules on micronutrient use efficiencies by cowpea.

Treatments	Mobilization Efficiency			Physiological Efficiency (q g^−1^)		
	Zn	Fe	Mo	Zn	Fe	Mo
M_0_F_0_	0.781 ^b^ ± 0.02	0.602 ^c^ ± 0.06	0.858 ^c^ ± 0.01	-	-	-
M_0_F_1_	0.867 ^a^ ± 0.03	0.639 ^bc^ ± 0.04	0.894 ^abc^ ± 0.04	0.368 ^bc^ ± 0.02	0.056 ^b^ ± 0.001	0.281 ^abc^ ± 0.02
M_0_F_2_	0.952 ^a^ ± 0.05	0.616 ^bc^ ± 0.01	0.709 ^d^ ± 0.04	0.355 ^bc^ ± 0.01	0.057 ^b^ ± 0.005	0.337 ^ab^ ± 0.04
M_0_F_3_	0.852 ^a^ ± 0.09	0.718 ^abc^ ± 0.06	0.874 ^bc^ ± 0.07	0.389 ^bc^ ± 0.01	0.057 ^b^ ± 0.005	0.268 ^abc^ ± 0.03
M_1_F_0_	0.726 ^b^ ± 0.1	0.728 ^ab^ ± 0.08	0.922 ^abc^ ± 0.11	0.499 ^ab^ ± 0.05	0.068 ^a^ ± 0.002	0.342 ^a^ ± 0.03
M_1_F_1_	0.855 ^ab^ ± 0.12	0.733 ^ab^ ± 0.11	0.919 ^abc^ ± 0.15	0.385 ^bc^ ± 0.09	0.056 ^b^ ± 0.004	0.254 ^abc^ ± 0.01
M_1_F_2_	0.848 ^a^ ± 0.07	0.728 ^ab^ ± 0.12	0.708 ^d^ ± 0.17	0.388 ^bc^ ± 0.12	0.057 ^b^ ± 0.001	0.325 ^ab^ ± 0.01
M_1_F_3_	0.890 ^a^ ± 0.02	0.761 ^a^ ± 0.11	0.932 ^abc^ ± 0.10	0.354 ^bc^ ± 0.11	0.056 ^b^ ± 0.001	0.246 ^abc^ ± 0.02
M_2_F_0_	0.738 ^b^ ± 0.01	0.733 ^ab^ ± 0.06	0.956 ^ab^ ± 0.07	0.572 ^a^ ± 0.12	0.069 ^a^ ± 0.004	0.350 ^a^ ± 0.09
M_2_F_1_	0.885 ^a^ ± 0.01	0.809 ^a^ ± 0.02	0.918 ^abc^ ± 0.09	0.359 ^bc^ ± 0.06	0.051 ^c^ ± 0.003	0.224 ^bc^ ± 0.11
M_2_F_2_	0.879 ^a^ ± 0.03	0.761 ^a^ ± 0.05	0.945 ^abc^ ± 0.05	0.361 ^bc^ ± 0.05	0.057 ^b^ ± 0.001	0.273 ^abc^ ± 0.12
M_2_F_3_	0.949 ^a^ ± 0.05	0.824 ^a^ ± 0.04	0.969 ^a^ ± 0.01	0.323 ^c^ ± 0.04	0.052 ^c^ ± 0.002	0.195 ^c^ ± 0.11
LSD (0.05)	0.16	0.12	0.09	0.15	0.001	0.11

M_0_: No molybdenum, M_1_: Molybdenum seed priming, M_2_: Molybdenum soil treatment, F_0_: No fertilizer application, F_1_: Fe application, F_2_: Zn application, F_3_: Fe+Zn application. The mean with a similar or dissimilar letter(s) was evaluated with the least significant difference (LSD) multiple range tests using a probability level of *p* ≤ 0.05 along with standard deviation.

## Data Availability

All data are available in the manuscripts.
